# Fermi states and anisotropy of Brillouin zone scattering in the decagonal Al–Ni–Co quasicrystal

**DOI:** 10.1038/ncomms9607

**Published:** 2015-10-07

**Authors:** V. A. Rogalev, O. Gröning, R. Widmer, J. H. Dil, F. Bisti, L. L. Lev, T. Schmitt, V. N. Strocov

**Affiliations:** 1 Department for Synchrotron Radiation and Nanotechnology (SYN), Swiss Light Source, Paul Scherrer Institute, Villigen CH-5232, Switzerland; 2 Department of Advanced Materials and Surfaces, EMPA, Swiss Federal Laboratories for Materials Science and Technology, Überlandstrasse 129, Dübendorf CH-8600, Switzerland; 3 Institute of Condensed Matter Physics, Ecole Polytechnique Fédérale de Lausanne, Lausanne CH-1015, Switzerland; 4 National Research Center Kurchatov Institute, Akademika Kurchatova Square 1, Moscow 123182, Russia

## Abstract

Quasicrystals (QCs) are intermetallic alloys that have excellent long-range order but lack translational symmetry in at least one dimension. The valence band electronic structure near the Fermi energy *E*_F_ in such materials is of special interest since it has a direct relation to their unusual physical properties. However, the Fermi surface (FS) topology as well as the mechanism of QC structure stabilization are still under debate. Here we report the first observation of the three-dimensional FS and valence band dispersions near *E*_F_ in decagonal Al_70_Ni_20_Co_10_ (d-AlNiCo) QCs using soft X-ray angle-resolved photoemission spectroscopy. We show that the FS, formed by dispersive Al *sp*-states, has a multicomponent character due to a large contribution from high-order bands. Moreover, we discover that the magnitude of the gap at the FS related to the interaction with Brillouin zone boundary (Hume–Rothery gap) critically differs for the periodic and quasiperiodic directions.

## Abstract (web summary)

Quasicrystals show long-range order but lack the symmetry of conventional crystals. Here, the authors study the bulk electronic structure of quasicrystals and their three-dimensional Fermi surface topology, providing links to the structural stability of these materials.

## Graphical abstract

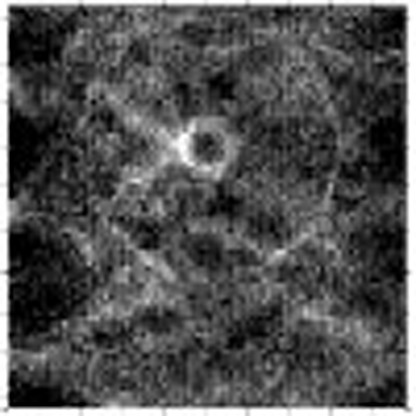

Decagonal quasicrystals (QCs) are extremely tempting structures to study due to the unique combination of the periodic and QP orders in the same crystal. The lattice of such materials can be obtained by appropriate projection of the periodic lattice in five-dimensional space to the three-dimensional (3D) space^1^. The real and the reciprocal space (**k**-space) lattice vectors of decagonal QCs thus can be denoted by five vectors^2^ (Fig. 1a), spanning a reciprocal lattice in aperiodic plane and a set of discrete maxima with 2*π/c* spacing in the periodic (00001) direction, where *c*≈4 Å is a lattice parameter for d-AlNiCo in this direction^3^. The corresponding low-energy electron diffraction (LEED) pattern from the clean tenfold surface of d-AlNiCo measured at electron beam energy *E*_e_=43 eV at room temperature is shown in Fig. 1b. The very existence of the decagonal QCs poses fundamental questions on how the duality between the periodic and QP orders manifests itself in electronic structure and how it is related to the stabilization of their unusual structure.

These questions have been explored in a number of theoretical articles^4^,^5^,^6^,^7^,^8^,^9^,^10^,^11^ and experimental works^12^,^13^,^14^,^15^,^16^,^17^,^18^,^19^. Angle-resolved photoemission spectroscopy (ARPES) experiments utilizing vacuum ultraviolet (VUV) radiation established the general electronic structure of d-AlNiCo, showing that in the binding energy *E*_b_ range far from *E*_F_ the Al *sp*-derived states have clear dispersive character and hence are effectively delocalized^20^,^21^. The observed dispersion character is close to free-electron like (FE like) with the effective mass ratio m*/*m*_e_≈1. Moreover, **k**-space locations of the *sp*-bands observed in ARPES correspond to the reciprocal lattice vectors **G** dominant in LEED in both periodic and QP directions in agreement with theoretical calculations^22^,^23^. The survival of FE-like dispersion in the aperiodic QC media is similar in nature to the incommensurate periodicities, found, for example, in one-dimensional Peierls compounds, where the same phenomena was observed in ARPES experiment^24^. The spectral weight in such structures is redistributed in accordance with the strength of the crystal potential and the structure factor in **k**-space and concentrated mainly on the FE-like parabola. The corresponding q-Brillouin zone (q-BZ) in the tenfold plane could be defined as a boundary between the central diffraction maximum and the most intense reflections in diffraction pattern^25^,^26^. According to the Spot Profile Analysis (SPA)-LEED study^27^, these high-intensity spots are distributed over a circle with a radius *k* of ∼2.7 Å^−1^. Along the periodic direction, the unit cell includes two non-equivalent adjacent aperiodic planes, nevertheless the effective size of the q-BZ along the periodic axis in **k**-space is doubled and is equal to 4*π/c* due to the non-symmorphic space group symmetry of d-AlNiCo (analogous, for example, to graphite^28^). On the basis of these principles, Fig. 1c shows the section of the 3D Fermi surface (FS) model of d-AlNiCo, where for clarity only the strongest reciprocal lattice vectors with *G*_||_={0 Å^−1^, 2.7 Å^−1^} at *G*_Z_={−1.54 Å^−1^, 0 Å^−1^, −1.54 Å^−1^} were chosen as the centres of FE-like spheres. The real FS could be obtained by increasing the set of **G**-vectors and taking into account the structure factors as well as all possible Bragg scattering effects at the q-BZ boundaries. The latter open multiple gaps at the *E*_F_ at certain points in **k**-space and transform the FS into a segmented FS that has a smaller surface area compared with the original one. Although the largest gaps are expected to appear at the first q-BZ boundary where the corresponding interaction potential Fourier components are the largest, due to the dense diffraction pattern the smaller gaps are expected to appear also in the higher q-BZs. Theoretical FSs were calculated *ab initio* for Y-AlNiCo structural model^6^ and for orthorhombic approximant to the decagonal phase^10^, both resulting in a highly anisotropic FS with a complex multiple-band structure.

However, all previously reported VUV-ARPES investigations did not succeed to establish the presence of dispersive states in the region near the *E*_F_, as well as the shape of the FS itself, which is important for understanding the mechanism of QC phase stabilization. Electronic structure calculations^4^,^29^,^30^ predicted that the density of states (DOSs) near *E*_F_ should have two features: a pseudogap at *E*_F_ with its characteristic energy scale of the order of 1 eV and much finer spikes and mini-pseudogaps with their characteristic scale of the order of tens of meV. The coexistence of these two types of the DOS features in decagonal QCs has been confirmed by various techniques^13^,^15^,^16^,^31^ including photoemission spectroscopy and scanning tunnel spectroscopy. The eV-scale pseudogap at *E*_F_ is believed to result from the destructive interference of Fermi states at the boundaries of the first q-BZ, which is referred as the Hume–Rothery (HR) mechanism, and/or the hybridization between transition metal (TM) *d*-states and Al *sp*-states^12^ (*sp-d* hybridization), while the relative weight of these two mechanism has not been defined yet. In turn, the meV-scale pseudogaps could appear due to the scattering of the Al *sp*-states at the boundaries of the higher-order q-BZs or due to the localized character of the electron wave function near *E*_F_. Since the QC structure provides a higher symmetry of the q-BZ comparing with the periodic lattice, the HR mechanism could significantly contribute to the formation of this large pseudogap (HR gap), thus being a driving force of QC phase stabilization. The HR electron concentration rule recently was revised theoretically using full potential linearized augmented planewave method for QC approximants involving sufficient number of the plain waves expansion^11^. At the same time, although the *sp-d* hybridization may indeed form a pseudogap at *E*_F_, it is less likely to stabilize the QC structure because, with the *d*-states being localized, the interaction integral between *sp*- and *d*-states
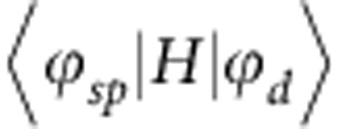
 (where *H* is the Hamiltonian) that contributes to the system total energy, responds only smoothly to a variance of the atomic positions.

In this study, we endeavour to observe the 3D FS of a bulk QC and to understand the role of pseudogap in stabilization mechanism of QC structure. We demonstrate that FS consists of dispersive Al *sp*-states with large contribution from high-order bands. We show that FS–BZ interaction is highly anisotropic, which results in a different magnitudes of HR gaps along QC and periodic directions.

## Results

### FS contours in QC planes

In ARPES, the HR gap should be clearly observed as bending of the band dispersions closer to *E*_F_^32^ at the q-BZ boundary, which leads to the formation of a pseudogap at *E*_F_. As a consequence, the FS should be not a sphere anymore but should break into a set of discrete pockets. To observe the 3D FS, we performed soft X-ray ARPES (SX-ARPES) experiments in the vicinity of *E*_F_ on decagonal AlNiCo QCs. Increase of the photoelectron mean free path *λ* at higher photoelectron energies make the SX-ARPES experiment more bulk sensitive^33^, which is essential for the QC samples because their surface stoichiometry may be distorted by structural defects and surface segregation. In addition, SX-ARPES reduces the final-state effects distorting the photoemission response of the valence states, in particular because the increase of *λ* scales down electron momentum broadening in the surface normal direction^34^, thus allowing accurate resolution of the native 3D bulk dispersions *E*(**k**). The tenfold (00001) surface of the sample was oriented normal to the analyser axis. We varied the surface perpendicular momentum *k*_z_ of the probed valence states through the photon energy *hv* and their **k**_**||**_ through the emission angle (refer also to the Methods section).

**Figure 1:**
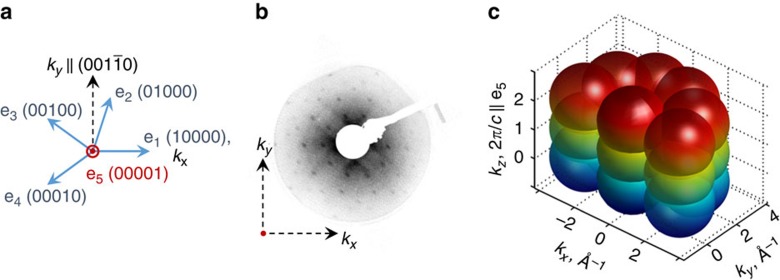
Quasicrystaline lattice basis and the model of electronic structure. (**a**) Set of reciprocal lattice base vectors. (**b**) LEED from the clean tenfold surface measured at electron beam energy *E*_e_=43 eV. (**c**) Section of the theoretical FS model in **k**-space in the simplest case that implies only central FE-like sphere and set of tenfold surrounding spheres with strongest diffraction intensity.

**Figure 2:**
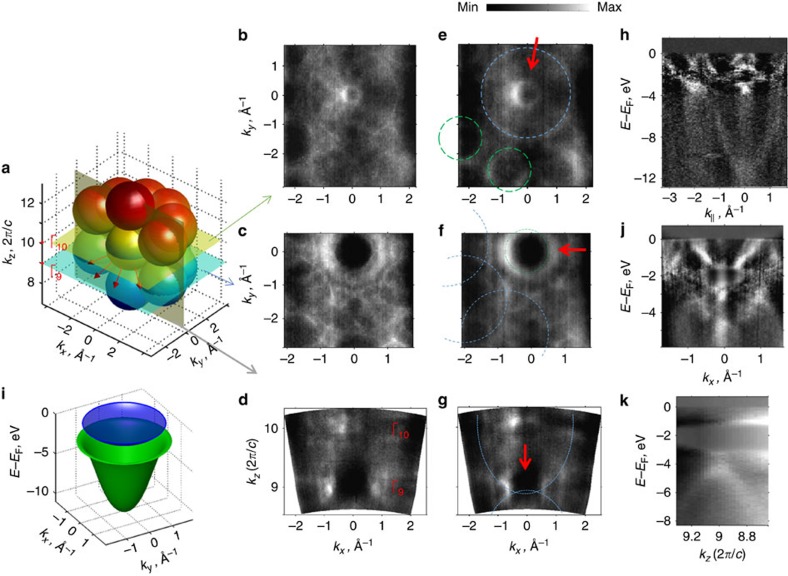
SX-ARPES data. (**a**) Sketch of the 3D FS observed in the current experiment with the planes corresponding to the different cuts represented in **b**–**g**. Red arrows denote the possible directions where eV-scale HR gap can appear in addition to the one observed along the periodical direction. (**b**–**d**) Constant binding energy maps (CEMs) corresponding to the FS cut acquired along the planes parallel to the sample surface at *k*_z_=10·(2*π/c*) denoted as Γ_10_-point (**b**) *k*_z_=9·(2*π/c*) denoted as Γ_9_-point (**c**) and perpendicular to the sample surface at *k*_*y*_=0 (**d**). (**e**–**g**) CEMs at *E*_b_=−0.6 eV acquired at the same planes as (**b**–**d**). The blue and green circles are the guide for the eye lines of Al *sp*-dispersion contours. (**h**,**j**,**k**) The ARPES intensity (logarithmic grayscale for **h** and **j**) maps *I*(*E*_b_, *k*_||_) measured along the directions shown with red arrows in **e**–**g**, respectively. (**i**) The 3D sketch of the *sp*-band dispersion parabola *E*_b_ (*k*_*x*_,*k*_*y*_) hybridized with the TM-*d* flat band.

The entire set of our experimental results is presented in Fig. 2. The sketch in Fig. 2a shows the main features of the experimentally observed 3D FS. It differs from the one presented in Fig. 1c because of the specific structure factor due to the non-symmorphic space group symmetry of d-AlNiCo. In addition, previous theoretical simulations of the photoemission from QCs suggested that there is a certain threshold for the relative strength of the lattice potential^35^, which significantly reduces the number of observable bands in the typical photoemission experiment. Photoelectron intensity maps at constant *E*_b_ (constant energy maps, CEMs) were acquired in planes in **k**-space shown in the sketch in Fig. 2a. These CEMs are reported for two different *E*_b_: corresponding to the FS (Fig. 2b–d) and halfway between *E*_F_ and TM *d*-bands maximum (Fig. 2e–g). Surprisingly, in CEM measured parallel to the surface at *k*_z_ in the Γ_10_-point (centre of the tenth BZ along the periodic axis, *hv*=875 eV), we observed a sharp and almost unaltered contour of FE-like Al *sp*-bands at *E*_F_ (Fig. 2b) with the Fermi momentum *k*_F_^0^≈1.57 Å^−1^ (shown as a blue circle at lower *E*_*b*_ in Fig. 2e). Apart from the circle-like contour of dispersive Al *sp*-states centred at surface parallel momentum **k**_**||**_=0, there is a small hole-like pocket at **k**_**||**_=0 with *k*_F_≈0.3 Å^−1^ and a manifold of surrounding electron-like pockets with *k*_F_^1^≈0.7 Å^−1^ with tenfold symmetry (green circles in Fig. 2e). The centres of these latter e-pockets are distributed on a circle with *k*≈2.7 Å^−1^, which corresponds to the radius of the 
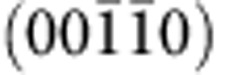
 reflections in the LEED pattern^27^. These directions belong to the set of twofold Δ axes rotated by *π*/10 from the ∑ axes, both having different intensity distribution patterns in diffraction^26^.

The ARPES intensity map at Γ_10_-point as a function of *E*_b_ and *k*_||_ (*I*(*E*_b_, *k*_||_)) along the axis, which belongs to the set of Δ axes is shown in Fig. 2h, where to enhance the figure contrast, we applied the background subtraction procedure (refer to the Methods section). The map appears to be quite complex in dispersion, especially in a region from *E*_F_ to 3 eV in binding energy, due to the contribution of the higher-order dispersions that originates from other points of the dense reciprocal lattice. Nevertheless, one can recognize that the FE-like parabola of Al *sp*-band is effectively divided in two energetic regions as a result of the *sp*-*d* hybridization, which is schematically shown in Fig. 2i in 3D (see the Methods section and Supplementary Fig. 3). In this figure, the lower part of the *sp*-band paraboloid *E*_b_ (*k*_*x*_,*k*_*y*_) is shown in green and the upper part near the *E*_F_ in blue. The high-*E*_b_ part of the Al *sp*-band in Fig. 2h appears to be more blurred than its near *E*_F_ counterpart due to the effects of electron momentum broadening in the surface normal direction and increased DOSs of TM *d*-bands at higher *E*_b_. In addition, the *sp-d* hybridization slightly reduces the value of *k*_F_ of the Fermi states, but does not affect the FS continuity. The survival of the FE-like FS contour and the absence of dispersion bands bending near *E*_F_ suggest that the energy scale of the pseudogap, induced by the HR mechanism in QP plane, is either vanishingly small or at least less than ∼70 meV, which is half of our actual experimental resolution. This value is significantly smaller than the reported value of the pseudogap of 1 eV order^31^.

Due to the non-symmorphic space group symmetry, completely different CEMs were observed at *k*_z_ in the Γ_9_-point (*hv*=720 eV), which would otherwise be identical to Γ_10_-point. Figure 2c,f reveals very intense central ring with *k*_F_^1^ (green circle in Fig. 2f) and less pronounced tenfold large circles with radius *k*_F_^0^ surrounding this circle (blue circles in Fig. 2f). The former ring corresponds to an electron-like pocket with band minimum around *E*_b_≈−2 eV as can be seen from *I*(*E*_b_, *k*_||_) acquired parallel to *k*_*x*_ through Γ_9_-point (Fig. 2j). Obviously, this electron-like pocket is a result of intersection of FE-like Fermi spheres formed by Al *sp*-states and centred at Γ-points at nearest even BZs along the periodic axis. The 2*π*/*c* shift of small and large e-pockets along the *k*_z_ (blue and green circles in Fig. 2e,f) is consistent with the results of the SPA-LEED experiment^27^, where the same shift in the periodicity along *k*_z_ of the full width at half maximum of the central and *k*≈2.7 Å^−1^ diffraction spots was observed. In addition, we note that the large circles that surround the central electron-like pocket in Fig. 2c do not show any significant gaps at *E*_F_ at intersection points and one can see the crossing arcs of these circles. It again confirms that the energy scale of the HR gap in QP plane (if present) should be in sub-eV scale.

### SX-ARPES along periodic direction

The FS character along the periodic direction is different however. In CEMs measured along the periodic axis (Fig. 2d,g), we observe sharp features at *k*_z_ at the Γ_10_- and Γ_9_-point, whereas between them we find a continuum of states without any well-defined dispersions. From the *I*(*E*_b_, *k*_||_) measured in the same plane along *k*_z_ near the Γ_9_-point (Fig. 2k), it becomes clear that there are also no dispersive Al *sp*-states from *E*_F_ to TM *d*-band at *k*_*x*_=0 near Γ_9_-point. This observation unveils the occurrence of the Bragg scattering of Al *sp*-states close to the *E*_F_ at the effective q-BZ boundary between two even Γ-points in periodic direction since the Fermi momentum *k*_F_^0^ matches 2*π/c*=1.54 Å^−1^—the periodicity along *k*_*z*_. The observed gap of the eV-scale reduces the integrated DOS of the occupied states at *E*_F_, minimizing the total energy of the system. Formation of such a gap is a keystone of HR mechanism and should indeed electronically stabilize the sample structure, but unexpectedly this eV-scale gap was observed only in periodical direction. With this observation, we can conclude that the HR mechanism in d-AlNiCo exhibits strong anisotropy: the eV-scale HR gap appears along the periodic direction, while the energy scale of the HR gap in QP planes has to be in the sub-eV range. We emphasize that these experimental results are consistent with the scanning tunnel spectroscopy studies, which found a manifold of locally dependent sets of small (20–50 meV) gaps in the occupied DOS of d-AlNiCo^16^,^31^. The theoretical calculations of the spectral density along the Δ axis of the 2D Penrose tiling^22^ also revealed the gaps near centre of the bands due to the localized character of the states rather than scattering at the q-BZ boundary.

It is interesting as well to correlate the observed energy scale anisotropy with well-studied vibrational spectra of the QCs. A larger pseudogap between the optic and acoustic excitation was observed in 1/1 approximant comparing with the QC counterpart^36^, suggesting the stronger Bragg-plane reflection for the periodic structure. On the other hand, inelastic neutron scattering^37^ did not reveal a significant anisotropy between the vibrational modes along the QP and periodic directions in d-AlNiCo.

We note that the previous VUV-ARPES data^21^, presented in the high-*E*_b_ sector of electronic structure, have certain differences respective to our SX-ARPES data in the number of the bands observed. Contributing to these differences can be, first, the highly different degree of surface sensitivity. Second, VUV-ARPES poorly defines the *k*_z_ component, which is intrinsically limited by non-FE character and *k*_z_ broadening of low-energy final states^34^. Finally, the final-state wavevector **k**_**f**_ in VUV-ARPES is much smaller than in SX-ARPES. If we treat the ARPES final state as a plane wave 
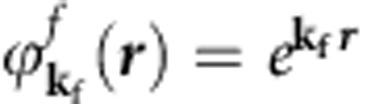
 with weak damping towards the crystal interior, the photoemission intensity appears essentially as a matrix element 
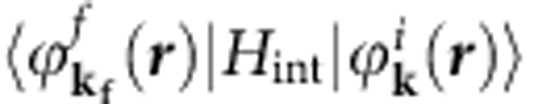
 between the initial state 
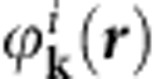
 transformed by the dipole operator *H*_*int*_ and the final state 
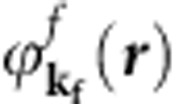
. Therefore, the VUV- and SX-photoemission probe different low- and high-**k** Fourier components, respectively, of the transformed initial state.

### Simulation of the FS contours in QC planes

**Figure 3:**
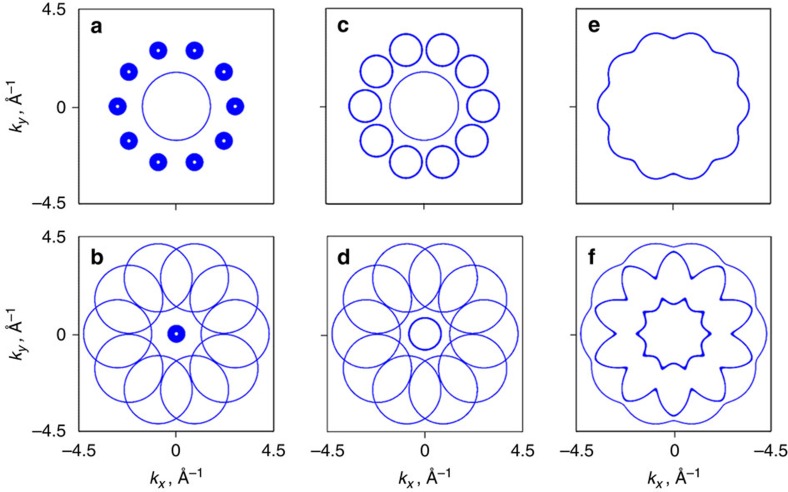
Simulation of FS contours. (**a**,**b**) Simulation results of the CEMs at *E*_F_ at *k*_z_ in the Γ_10_- and Γ_9_-points, respectively, in (*k*_*x*_,*k*_*y*_) plane without the Bragg scattering at the q-BZ boundaries. (**c**,**d**) Same as **a** and **b**, but with the Bragg scattering allowed at the q-BZ boundaries only in periodical direction. (**e**,**f**) Same as **a** and **b**, but with the Bragg scattering allowed at the q-BZ boundaries in both QP and periodical directions.

To simulate the stronger in-QP-plane HR gap at the *E*_F_, we utilized a 3D theoretical model that consists of the single near-*E*_F_ part of Al *sp-d* hybridized bands (blue part of the hybridized *sp-d* band in Fig. 2i) centred at the same spots in **k**-space as observed in the present experiment (see the Methods section). We simulated the FS cuts along **k**_**||**_ at *k*_z_ in the even and odd Γ-points with and without Bragg scattering at the q-BZ boundaries in the QP and periodic directions (Fig. 3). These cuts reflect the measured CEMs shown in Fig. 2b,c with different parameters of the scattering strength at q-BZ boundaries taken into account. The value of the scattering strength was chosen to provide the HR gap of eV-order for the QP and periodic directions to match the experimentally observed value of the HR gap along the *k*_*z*_ axis (Fig. 2k). Corresponding dispersion relations *E*_b_ (*k*_*x*_,*k*_*y*_=0) for different cases shown in Fig. 3a–f are presented in Supplementary Fig. 4a–f, respectively. From Fig. 3, it becomes clear that the experimentally observed FS (Fig. 2b,c) corresponds better to the case when the eV-scale HR gap is present along the periodic direction only (Fig. 3c,d), while the same HR gap present in both periodic and QC planes should significantly alter the FS contours (Fig. 3e,f).

## Discussion

It is commonly believed that there are two main alternative mechanisms for stabilization of the QP phase: the energy stabilization with the QC ground state and the entropy stabilization with a crystalline phase at low temperature^38^. The observed energy scale of the HR gap along the QC direction is significantly lower or even vanishing compared with the one along periodic direction, suggesting that the in-QC-plane (aperiodic) structure does not lead to a significant Peierls-type energy contribution to the structural stability. On the other hand, there could be an additional contribution to the 3D structure stabilization via the HR gaps along the ‘off-symmetry' directions shown in Fig. 2a with red arrows. In fact, the distance in **k**-space between centres of FE-like sphere at *k*_||_=0 at Γ_10_-point and tenfold FE-like spheres observed on a radius *k*_||_=2.7 Å^−1^ at Γ_9_-point (Fig. 2a) matches the diameter of these spheres *d*=2·*k*_F_^0^, thus enabling the possibility for the eV-scale HR gap to appear. For the icosahedral QC, due to the presence of only aperiodic planes, we suppose that the energy scale of HR gaps is more likely to be uniform.

We note that the direct observation of the FS–BZ scattering anisotropy has only been possible by the virtue of accurate definition of 3D electron momentum achieved with SX-ARPES. This has been essential to disentangle the electron dispersions in the periodic and aperiodic planes of d-AlNiCo. With our results, we hope to stimulate the advanced theories capable of explaining the observed anisotropy from first principles.

## Methods

### Samples and SX-ARPES measurements

Single crystals of decagonal AlNiCo with the bulk composition Al_70_Ni_20_Co_10_ were grown by means of Czochralski method. Slow growth out of the melt over 300 h at the solidification temperature ensured high sample quality. The (00001)-oriented surface (tenfold) of AlNiCo was cleaned by cycles of Ar^+^ ion sputtering and subsequent annealing up to 750 °C leading to sharp tenfold symmetric LEED (Fig. 1b) and the single metallic component in Al*2p* core level photoemission spectrum. The measurements were conducted at the ADRESS beamline at Swiss Light Source synchrotron facility, Paul Scherrer Institute, Switzerland, using photon energy range *hv*=315–1,000 eV and different polarizations of incident X-rays^33^. The temperature of the samples during the measurements was around 12 K. The combined (beamline+analyser) energy resolution was down to 70 meV. The analyser entrance slit was oriented along the X-ray beam incident on the sample at a grazing incidence angle of 20° (for further experimental details see ref. 33).

### Data processing

Due to the absence of the translational periodicity in the QC lattice and a small Al-*sp* cross-section at the given photon energies, the quasiparticle peaks in valence band maps are rather weak and broadened along the momentum axis. Nevertheless, the raw data itself allowed us to recognize immediately the Al *sp*-bands at main high-symmetry points (Supplementary Fig. 1a). To increase the quality of the observed dispersions, we applied several procedures to the measured ARPES intensity maps *I (E*b*, k*_||_). First, the background from the charge-coupled device was taken into account and subtracted from the raw image. At the second step, the non-dispersive intensity from TM *d*-states was suppressed by subtraction of the substantially smoothed in momentum directions ARPES intensity map *I* (*E*_b_, *k*_||_) from the original raw images. The resulting processed image is shown in Supplementary Fig. 1b. The subtraction of the strongly smoothed image was also applied for the contrast enhancement in constant binding energy maps (CEMs).

### Polarization dependencies and *sp-d* hybridization

All the data presented in the paper were measured using the *p*-polarized light. The valence band maps measured at Γ_9_-point in *s*-polarized and *p*-polarized light are shown in Supplementary Fig. 2a,b, respectively. Interestingly, the photoemission intensity from the dispersive Al states vanishes in *s*-polarized light (anti-symmetric in the given experimental geometry) confirming the symmetric nature of these states with respect to the measurement plane. The degree of hybridization between Al *sp*- and TM *d*-states could be estimated from the angle integrated spectra measured at Γ-points at *k*_z_=6·(2*π/c*) (*hv*≈315 eV) and *k*_z_*=*10·(2*π/c*) (*hv*≈875 eV) shown in Supplementary Fig. 1c. Two spectra almost coincide however the relative cross-section of Al *sp*-states to TM *d*-states increases significantly with the photon energy^39^. Moreover, the *sp-d* hybridization is clearly observed in Fig. 2h as a bending of Al *sp*-bands closer to the original TM *d*-states to avoid crossing. This effectively divides the dispersion of Al *sp*-states into two regions with different *E*_b_ range (Fig. 2i). As a consequence, at Γ_9_-point, there is an additional spectral weight at *E*_b_∼4.5 eV near *k*_*x*_=0 that comes from higher *E*_b_ part of the Al *sp*-dispersion at Γ_10_- and Γ_8_-points (Supplementary Fig. 2a).

### The theoretical model and simulation results

The model to simulate the dispersion of the *sp*-bands in the d-AlNiCo structure is based on multiple Bragg reflections of the nearly free electrons on a set of the major components of the reciprocal quasi-lattice {**G**_**i**_}. For a given **k**-vector, the corresponding tight binding Hamiltonian can be written as follows:


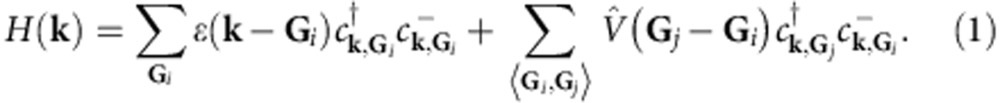


Here 
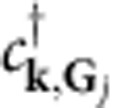
 and 
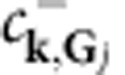
 are denoting the creation and annihilation operators, respectively, 
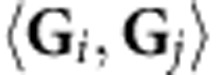
 denotes the pairwise sum for 
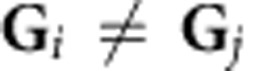
 and 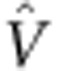 represents the valence electron potential in reciprocal space. The kinetic energy term of the model Hamiltonian was chosen to match the lower-*E*_b_ part of the hybridized *sp-d* band (blue curve in Supplementary Fig. 3) with *k*_F_^0^=1.57 Å^−1^, which was fitted with the polynomial equation:


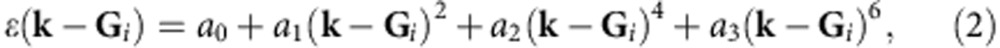


where *a*_0_=−1.34, *a*_1_=0.25, *a*_2_=−0.03 and *a*_3_=0.06. The selection of a finite set of {**G**_**i**_} for the calculation was made according to the most intense spots in the LEED. The set of {**G**_**i**_} consists of an alternating stack in *k*_*z*_ at (*k*_*x*_,*k*_*y*_)=(0,0) with *k*_*z*_=−3.08, 0 and 3.08 Å^−1^ and a tenfold rings with |**k**|=2.71 Å^−1^ at *k*_*z*_=−4.62, −1.54, 1.54 and 4.62 Å^−1^. This gives a total of 43 reciprocal vectors.

The strength of the potential 
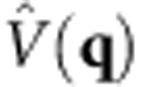
 was modelled as a Gaussian centred at k_0_:


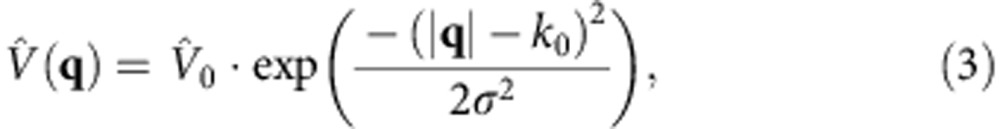


where 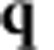 is the difference between pair of **G**-vectors, 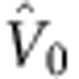 stands for the strength of the potential, *k*_0_=2.7 Å^−1^ and *σ*=0.84 Å^−1^. The parameter values were chosen to induce a gap of 1-eV order at *E*_F_ at q-BZ boundaries. Figure 3a,b shows the simulation result for the CEMs at *E*_F_ at Γ_10_- and Γ_9_-points along the *k*_*z*_, respectively, with the strength of the potential 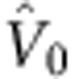 set to zero, which corresponds to the absence of the Bragg scattering at the q-BZ boundaries. Figure 3c,d shows the simulation result when the strength of the potential 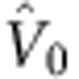 had a value of −0.7 eV for the periodic direction, while for the QP direction it was set to zero. This case corresponds to the occurrence of the Bragg scattering at the q-BZ boundaries along the periodic direction only. Finally, Fig. 3e,f shows the simulation result when the strength of the potential 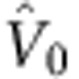 was set to −0.7 eV for both QP and periodic direction. The corresponding cuts *E*_b_ (*k*_*x*_,*k*_*y*_=0) for different cases shown in Fig. 3a–f are presented in Supplementary Fig. 4a–f, respectively.

## Additional information

**How to cite this article:** Rogalev, V. A. *et al*. Fermi states and anisotropy of Brillouin zone scattering in the decagonal Al–Ni–Co quasicrystal. *Nat. Commun*. 6:8607 doi: 10.1038/ncomms9607 (2015).

## Supplementary Material

Supplementary Information Supplementary Figures 1-4

## References

[b1] Yamamoto A. Crystallography of quasiperiodic crystals. Acta Crystallogr. A 52, 509–560 (1996) .

[b2] Steurer W., Haibach T., Zhang B., Kek S. & Lück R. The structure of decagonal Al_70_Ni_15_Co_15_. Acta Crystallogr. B B49, 661–675 (1993) .

[b3] Steurer W. Twenty years of structure research on quasicrystals. Part I. Pentagonal, octagonal, decagonal and dodecagonal quasicrystals. Z. Kristallogr. 219, 391–446 (2004) .

[b4] Trambly de Laissardière G. & Fujiwara T. Electronic structure and transport in a model approximant of the decagonal quasicrystal Al-Cu-Co. Phys. Rev. B 50, 9843–9850 (1994) . 10.1103/physrevb.50.9843 9975064

[b5] Krajčí M., Hafner J. & Mihalkovič M. Atomic and electronic structure of decagonal Al-Ni-Co alloys and approximant phases. Phys. Rev. B 62, 243–255 (2000) . 10.1103/physrevb.51.17355 9978763

[b6] Smontara A., Smiljanić I., Ivkov J. & Stanić D. Anisotropic magnetic, electrical, and thermal transport properties of the Y-Al-Ni-Co decagonal approximant. Phys. Rev. B 78, 104204 (2008) .

[b7] Inukai M., Soda K., Kato M., Yagi S. & Yokoyama Y. Cluster study of Al–Co–Ni decagonal quasicrystal. Z. Kristallogr. 223, 851–854 (2008) .

[b8] Mizutani U. . e/a Determination for the transition metal element TM in Al–Cu–TM–Si (TM=Fe and Ru) approximants and B2-compounds by means of the FLAPW-Fourier method. Z. Kristallogr. 224, 17–20 (2009) .

[b9] Komelj M., Ivkov J., Smontara A. & Gille P. Origin of the Hall-coefficient anisotropy in the Y–Al–Ni–Co periodic approximant to the decagonal phase. Solid State Commun. 149, 515–518 (2009) .

[b10] Dolinšek J. . Anisotropic magnetic and transport properties of orthorhombic Al_13_Co_4_. Phys. Rev. B 79, 184201 (2009) .

[b11] Mizutani U., Inukai M., Sato H. & Zijlstra E. S. Hume-Rothery stabilization mechanism and e/a determination in MI-type Al–Mn, Al–Re, Al–Re–Si, Al–Cu–Fe–Si and Al–Cu–Ru–Si 1/1-1/1-1/1 approximants – a proposal for a new Hume-Rothery electron concentration rule. Philos. Mag. 92, 1691–1715 (2012) .

[b12] Belin-Ferré E. Electronic structure of quasicrystalline compounds. J. Non-Cryst. Solids 334-335, 323–330 (2004) .

[b13] Stadnik Z., Purdie D., Garnier M. & Baer Y. Electronic structure of quasicrystals studied by ultrahigh-energy-resolution photoemission spectroscopy. Phys. Rev. B 55, 10938–10951 (1997) . 10.1103/PhysRevLett.77.1777 10063169

[b14] Okada J. & Watanabe Y. Electron momentum distribution of decagonal Al_72_Ni_12_Co_16_ studied by Compton scattering. J. Phys. Condens. Matter 14, L43–L48 (2002) .

[b15] Suzuki T. . Electronic structure of the topmost tenfold surface of decagonal Al-Ni-Co quasicrystal. Phys. Rev. B 72, 115427 (2005) .

[b16] Mäder R., Widmer R., Gröning P. & Deloudi S. High-resolution scanning tunneling microscopy investigation of the (12110) and (10000) two-fold symmetric d-Al-Ni-Co quasicrystalline surfaces. Phys. Rev. B 80, 035433 (2009) .

[b17] Soda K. . Spectroscopic study of Ni-rich Al–Co–Ni quasicrystal. Philos. Mag. 91, 2510–2518 (2011) .

[b18] Nayak J. . Bulk electronic structure of quasicrystals. Phys. Rev. Lett. 109, 216403 (2012) . 23215602 10.1103/PhysRevLett.109.216403

[b19] Nayak J. . Bulk electronic structure of Zn-Mg-Y and Zn-Mg-Dy icosahedral quasicrystals. Phys. Rev. B 91, 235116 (2015) .

[b20] Rotenberg E., Theis W., Horn K. & Gille P. Quasicrystalline valence bands in decagonal AlNiCo. Nature 406, 602–605 (2000) . 10949295 10.1038/35020519

[b21] Rotenberg E., Theis W. & Horn K. Electronic structure investigations of quasicrystals. Prog. Surf. Sci. 75, 237–253 (2004) .

[b22] Niizeki K. & Akamuatsu T. The reciprocal space properties of the electronic wave functions of the Penrose lattice. J. Phys. Condens. Matter 2, 7043–7047 (1990) .

[b23] Niizeki K. & Akamatsu T. Special points in the reciprocal space of an icosahedral quasi-crystal and the quasi-dispersion relation of electrons. J. Phys. Condens. Matter 2, 2759–2771 (1990) .

[b24] Voit J., Perfetti L., Zwick F. & Berger H. Electronic structure of solids with competing periodic potentials. Science 290, 501 (2000) . 11039927 10.1126/science.290.5491.501

[b25] Niizeki K. A classification of special points of quasilattices in two dimensions. J. Phys. A Math. Gen. 22, 4281–4293 (1989) .

[b26] Niizeki K. The diffraction pattern of a non-Bravais-type quasicrystal with application to a decagonal quasicrystal. J. Phys. Soc. Jpn 63, 4035–4043 (1994) .

[b27] Gierer M., Mikkelsen A., Gräber M., Gille P. & Moritz W. Quasicrystalline surface order on decagonal Al_72.1_Ni_11.5_Co_16.4_: An investigation with spot profile analysis LEED. Surf. Sci. 463, L654–L660 (2000) .

[b28] Chung D. D. L. Review graphite. J. Mater. Sci. 37, 1–15 (2002) .

[b29] Fujiwara T. & Yokokawa T. Universal pseudogap at Fermi energy in quasicrystals. Phys. Rev. Lett. 66, 333–336 (1991) . 10043779 10.1103/PhysRevLett.66.333

[b30] Smith A. & Ashcroft N. Pseudopotentials and quasicrystals. Phys. Rev. Lett. 59, 1365–1368 (1987) . 10035213 10.1103/PhysRevLett.59.1365

[b31] Mäder R., Widmer R., Gröning P., Steurer W. & Gröning O. Correlating scanning tunneling spectroscopy with the electrical resistivity of Al-based quasicrystals and approximants. Phys. Rev. B 87, 075425 (2013) .

[b32] Trambly de Laissardière G., Nguyen-Manh D. & Mayou D. Electronic structure of complex Hume-Rothery phases and quasicrystals in transition metal aluminides. Prog. Mater. Sci. 50, 679–788 (2005) .

[b33] Strocov V. N. . Soft-X-ray ARPES at the Swiss Light Source: From 3D Materials to Buried Interfaces and Impurities. Synchrotron Radiat. News 27, 31–40 (2014) .

[b34] Strocov V. N. Intrinsic accuracy in 3-dimensional photoemission band mapping. J. Electron Spectrosc. Relat. Phenom. 130, 65–78 (2003) .

[b35] Rotenberg E., Theis W. & Horn K. Model simulations of momentum-resolved photoemission from quasicrystals. J. Alloys Compd. 342, 348–351 (2002) .

[b36] De Boissieu M. . Lattice dynamics of the Zn-Mg-Sc icosahedral quasicrystal and its Zn-Sc periodic 1/1 approximant. Nat. Mater. 6, 977–984 (2007) . 17982466 10.1038/nmat2044

[b37] Dugain F. . Inelastic neutron scattering study of the dynamics of the AlNiCo decagonal phase. Eur. Phys. J. B 7, 513–516 (1999) .

[b38] Steurer W. & Deloudi S. Decagonal quasicrystals - What has been achieved? C. R. Phys. 15, 40–47 (2014) .

[b39] YehJ. & LindauI. Atomic subshell photoionization cross sections and asymmetry parameters:1 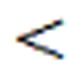 Z 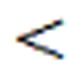 103. At. Data Nucl. Data Tables 32, 1–155 (1985) .

