# Supplementary material for: Fermi states and anisotropy of Brillouin zone scattering in the decagonal Al–Ni–Co quasicrystal
Source: Nat Commun. 2015 Oct 7;6:8607. doi: 10.1038/ncomms9607 (PMC4633949; doi:10.1038/ncomms9607)
Supplement: Supplementary Information — Supplementary Figures 1-4 [file ncomms9607-s1.pdf]

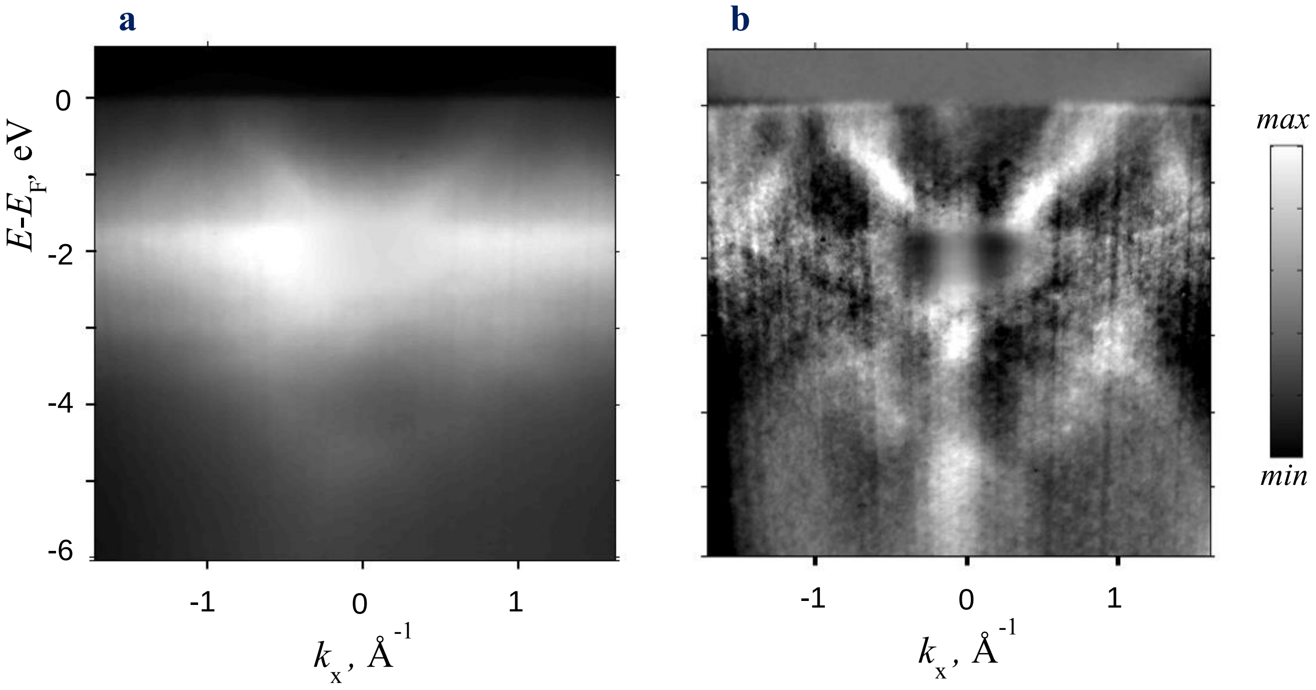

Supplementary Figure 1. The ARPES intensity map $I(E_b, k_\parallel)$ at k_z in the Γ_9-point: as measured (**a**) and processed (**b**) images.

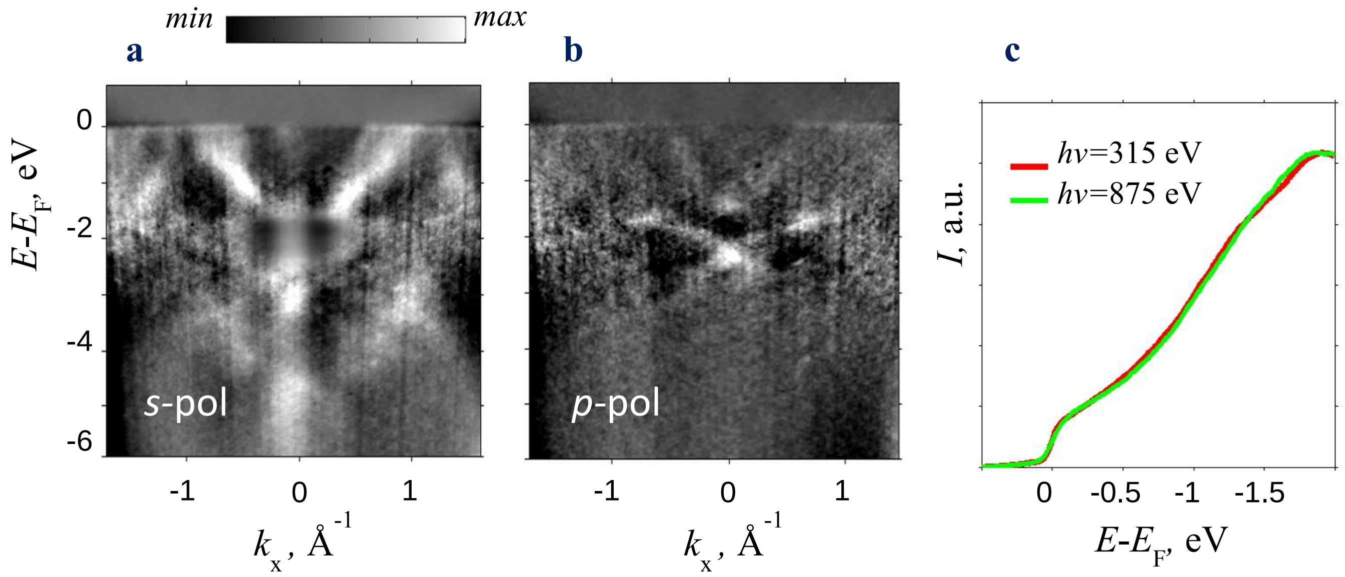

Supplementary Figure 2. The ARPES intensity map $I(E_b, k_\parallel)$ measured at k_z in the Γ_9-point along $\mathbf{k_x}$ with the s-polarized light (**a**) and p-polarized light (**b**). Angle integrated spectra measured at Γ-points at $k_z = 3\cdot(2\pi/c)$ ($h\nu \approx 315$ eV) and $k_z = 5\cdot(2\pi/c)$ ($h\nu \approx 875$ eV) (**c**).

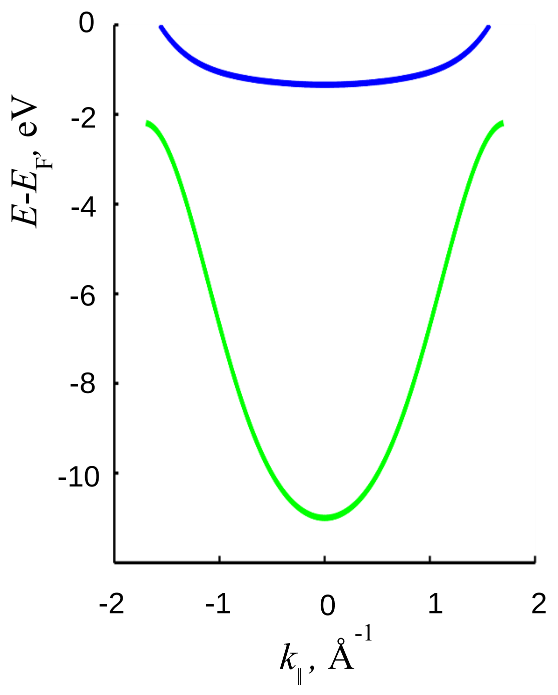

Supplementary Figure 3. The simulated hybridized *sp*-*d* bands with k_F^0=1.57 Å^{-1} with the lower-E_b part shown in blue color and the higher-E_b part shown in green.

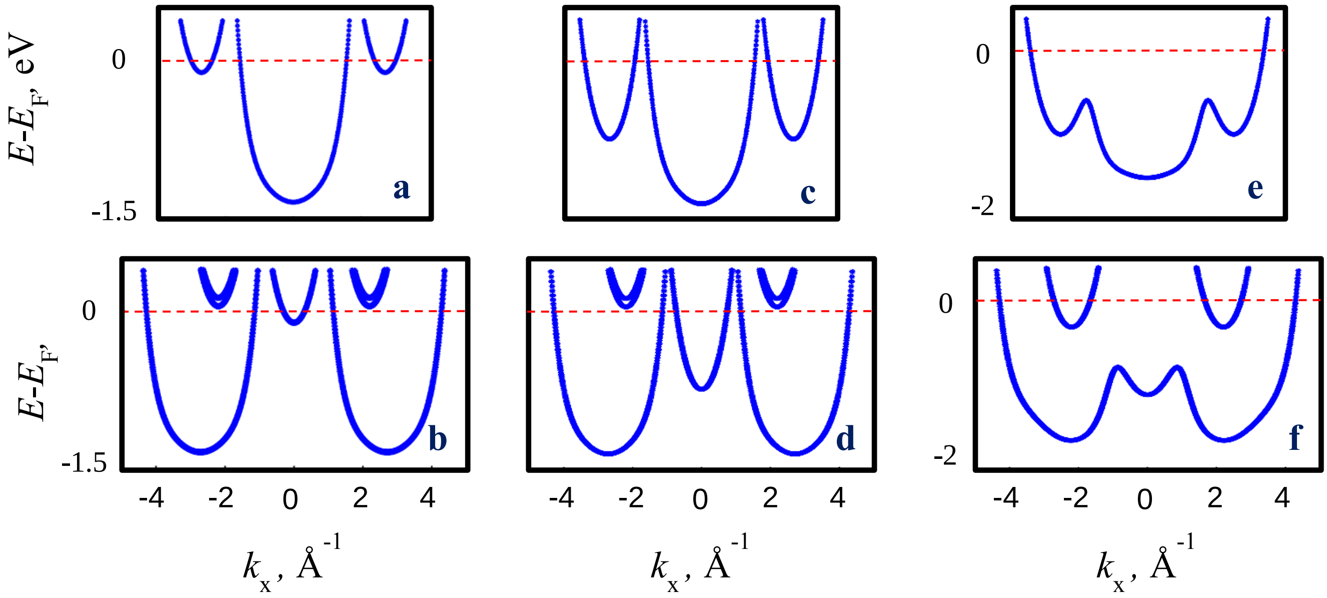

Supplementary Figure 4.a-f. Simulated dispersions E_b (k_x, k_y=0) of the lower-E_b part of hybridized *sp-d* bands near E_F that corresponds to the Fig.3a-f.
